# Interneuronal correlations at longer time scales predict decision signals for bistable structure-from-motion perception

**DOI:** 10.1038/s41598-019-47786-1

**Published:** 2019-08-07

**Authors:** D. F. Wasmuht, A. J. Parker, K. Krug

**Affiliations:** 1 Department of Physiology, Anatomy and Genetics, University of Oxford, Oxford, OX1 3PT United Kingdom; 2 Department of Experimental Psychology, University of Oxford, Oxford, OX1 3UD United Kingdom; 3 Institute of Biology, Otto-von-Guericke University, Leipzigerstr. 44, 39120 Magdeburg, Germany

**Keywords:** Extrastriate cortex, Perception

## Abstract

Perceptual decisions are thought to depend on the activation of task-relevant neurons, whose activity is often correlated in time. Here, we examined how the temporal structure of shared variability in neuronal firing relates to perceptual choices. We recorded stimulus-selective neurons from visual area V5/MT while two monkeys (*Macaca mulatta*) made perceptual decisions about the rotation direction of structure-from-motion cylinders. Interneuronal correlations for a perceptually ambiguous cylinder stimulus were significantly higher than those for unambiguous cylinders or for random 2D motion during passive viewing. Much of the difference arose from correlations at relatively long timescales (hundreds of milliseconds). Choice-related neural activity (quantified as choice probability; CP) for ambiguous cylinders was positively correlated with interneuronal correlations and was specifically associated with their long timescale component. Furthermore, the slope of the long timescale - but not the instantaneous - component of the correlation predicted higher CPs towards the end of the trial i.e. close to the decision. Our results suggest that the perceptual stability of structure-from-motion cylinders may be controlled by enhanced interneuronal correlations on longer timescales. We propose this as a potential signature of top-down influences onto V5/MT processing that shape and stabilize the appearance of 3D-motion percepts.

## Introduction

Understanding the relationship between the firing of cortical neurons and the formation of perceptual decisions has been one of the central goals in systems neuroscience^1,2^. Investigations in the extrastriate, visual area V5/MT of macaque monkeys have played an important role in this endeavour. V5/MT neurons show selective tuning for motion direction^3^ and binocular disparity^4,5^ and causally contribute to the percept of both motion and binocular depth^6,7^. It was first observed in V5/MT that neuronal activity correlated with the percept reported by the monkey on a trial-y-trial basis, even when the stimulus exciting the given neuron was fixed^8^. A measure of this correlation is choice probability (CP), which refers to the probability that an independent observer correctly predicts an upcoming choice, solely based on the neuronal spiking activity. While significant CPs have been described in a number of sensory cortical areas and using different perceptual tasks^8–18^, their origin is debated^19–21^.

Determination of the origins of choice-related firing in cortical sensory areas will shed light on the emergence and sequence of decision-making processes. In a simple feed-forward model, variability in choice is directly caused by variability in the evoked response of sensory neurons, captured in their CP. Early theoretical models considering this interpretation, suggest that a decision is determined by comparing the CPs of pools of oppositely tuned neurons^8,22,23^. This bottom-up model can be contrasted with a top-down model, in which choice-related variability in the sensory response represents decision-linked modulatory feedback. In this case, the observed CP might be a reflection of latent internal states, such as attention or intrinsic biases, which may affect decision-making at multiple stages^16,20,21,24–27^.

Theoretical and experimental evidence directly links CP to shared response variability among sensory neurons^22,28–31^, also termed noise- or spike count correlation (rSC). Specifically, a neuron that is not directly linked to a decision process might show a significant CP simply because it is correlated with neurons that are. Hence, CP observed in a neuron depends both on the neurons relative read-out weight, i.e. contribution to the decision, and the structure of its correlations with other neurons^22,30^. A variety of mechanisms may generate such shared interneuronal correlations in sensory cortical areas^29,32–34^. First, correlations might arise between neurons that receive common input from sensory afferents. On this view, correlations should be largely driven in a bottom-up fashion, perhaps through factors such as eye movements^35^ or stochastic fluctuations in the stimulus^36^. Secondly, the common signal could arise through recurrent local connectivity^37,38^ including long range horizontal connections between neurons of similar tuning properties^39^. Such ideas find support in the spatial structure of interneuronal correlations since they become larger with physical proximity and similarity in stimulus preference of neuronal pairs^36,39–41^. Thirdly, there also exists substantial evidence for a more global and flexible source for correlations, possibly provided by top-down efferent projections (i.e. feedback connections)^19,42^. This type of correlation is associated with processes downstream of the sensory area such as feature-based attention^43–45^ or task instructions related to a decision^24,28^.

Turning to CPs, the question of their emergence therefore becomes linked in part to determining the source and structure of the underlying interneuronal correlations. For identification of potential sources for noise correlations, it is crucial to examine the timescales on which those correlations unfold. Noise correlations typically act on timescales in the range of a few tens to hundreds of milliseconds^36^. Although measured timescales vary between studies^32,46^, it is thought that feed-forward, i.e. bottom-up correlations, caused by the external stimulus, act on a faster timescale than those induced by top-down, i.e. feedback signals^47,48^, but see^49^. A recent study by Wimmer *et al*.^21^ used a hierarchical network model consisting of a sensory and a higher order integration circuit, which was trained on the classic random dot motion task. They found that CP measured in the sensory layer could be explained by a combination of a fast-acting bottom-up signal and a slower top-down component from the integration layer. The latter was proposed to increase over time injecting additional correlations into the sensory layer, which led to higher measurable CPs in those neurons.

Here, we recorded from V5/MT neurons while monkeys judged the rotation direction of a structure-from-motion (SFM) cylinder. Perceptual interpretation of this stimulus requires integration of motion direction and binocular depth information^7^. Previous studies have shown that the perceptually bistable version of the cylinder stimulus (i.e. the ambiguous cylinder) is associated with relatively large CPs when compared to the motion discrimination task (0.67 vs. 0.56 respectively)^8,10^. This might reflect a strong cylinder percept even in the ambiguous case and differences in population coding for visual motion and stereo depth. However, this comparison is taken across different studies in different laboratories with animals trained on different tasks. Applying an interleaved design including cylinders with varying disparity and random motion stimuli, we investigated spike count correlations, the timescale of the underlying neuronal interactions, and their relationship to the time-course of choice related activity, measured as CP. We find that the ambiguous version of the cylinder is associated with high interneuronal correlations at longer timescales. Furthermore, the long timescale component of these correlations predicts the large CP values observed for the ambiguous cylinder. We propose that this distinct correlation signature may reflect a top-down influence onto V5/MT processing, which could convey and stabilize the perceptual appearance of bistable visual stimuli.

## Results

Two monkeys (*Macaca mulatta*) performed a perceptual decision-making task, judging the rotation direction of structure-from-motion (SFM) cylinder stimuli that had different degrees of ambiguity (defined by the specific conjunction of motion direction and binocular disparity). Cylinder stimuli were pseudo-randomly interleaved with random dot motion stimuli with zero net motion, for which no decision was required. While monkeys were performing the cylinder task, we recorded electrical activity from 88 sites (after exclusion, see Methods) in cortical area V5/MT with single electrodes. Visual stimuli were optimized online for the disparity- and direction-selectivity of the recorded single neuron. Single unit activity (SU) was isolated from its surrounding multi-unit activity (MU) to quantify interneuronal correlations in response to the different conditions. SUs included in this study were tuned for binocular disparity and direction of motion (p < 0.05, *ANOVA*). Some of the SU data have been previously reported^10^. The central aim of this study was to uncover interneuronal correlation structures underlying the processing of bistable SFM stimuli during a perceptual decision-making task.

### Higher spike count correlations for the bistable cylinder stimulus than for random motion

**Figure 1:**
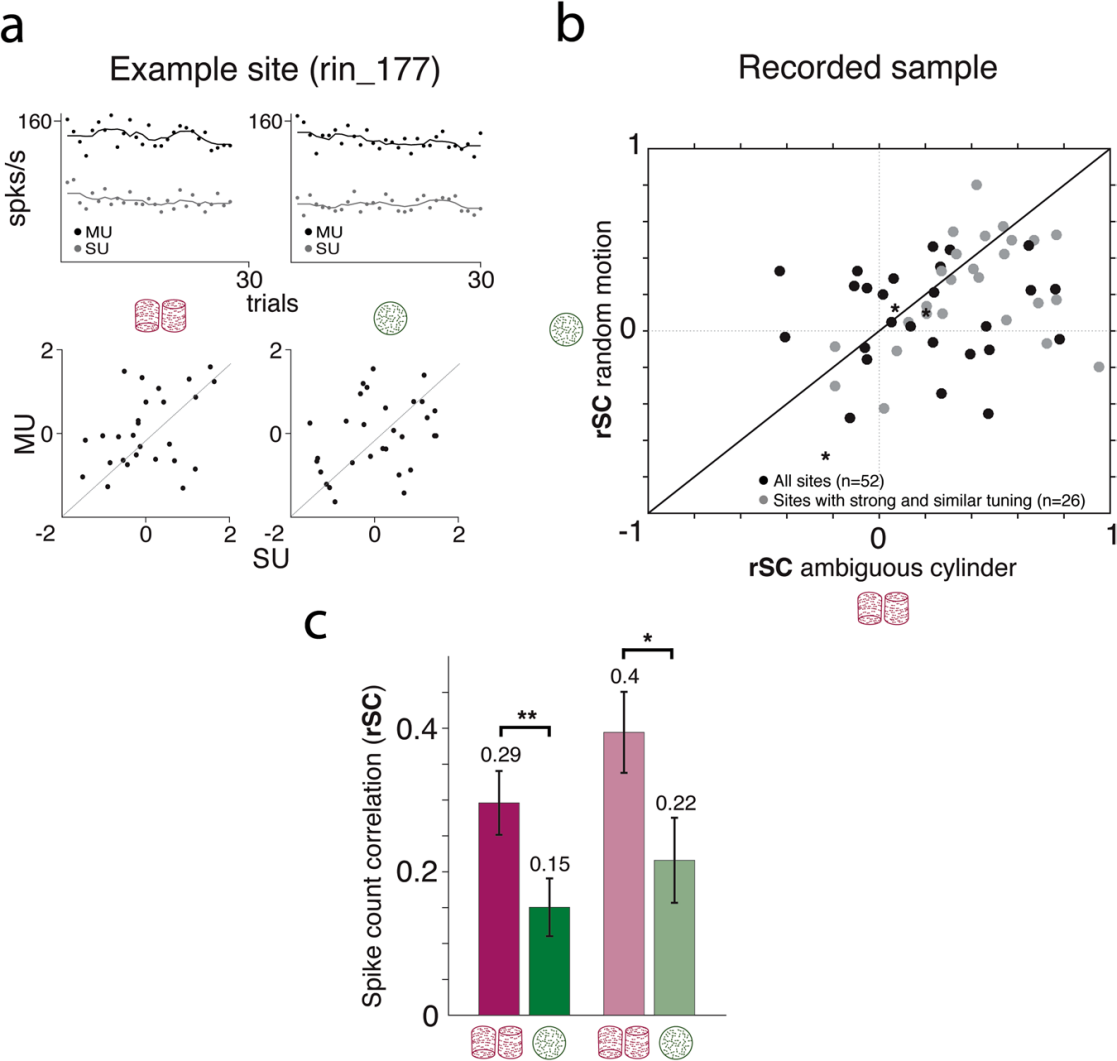
Comparing spike count correlations for the ambiguous cylinder and random dot stimulus. (**a**) Example site (rin_161); recorded activity in response to an ambiguous cylinder stimulus (left; indicated by the purple schemata) and random motion (right, green schemata). Upper two panels: Dots represent trialwise average firing rate (spks/s) of the SU (grey) and MU (black) recorded on the same electrode. Solid lines indicate the running mean computed over 11 trials centred on the current trial. Lower two panels: Scatter plots of SU and MU z-scores for each stimulus. The Pearson correlation between the z-scores gives the rSC (left; rSC = 0.43; right: rSC = 0.29). (**b**) The rSC values for the random motion stimulus (y-axis) are plotted against the rSC values for the ambiguous cylinder (x-axis) from the same sites. Data points show all sites that were recorded with both stimuli pseudo-randomly interleaved (n = 52). Grey dots show sites that showed strong and matching SU and MU tuning (n = 26 of 52) (see Methods). (**c**) Bar plot comparing the average rSC between the two stimuli; ambiguous cylinder (purple) and random motion (green). Bright bars show average values for all sites (n = 52); pale bars show average values for sites with strong and matching SU and MU tuning (n = 26 of 52). *p < 0.05, **p < 0.01; *t-test on Fisher’s z*. Error bars indicate the s.e.m.

First, we directly compared interneuronal correlations in response to ambiguous cylinder stimuli (zero disparity; evoking a bistable percept) to those evoked by a random dot stimulus^50,51^ in order to assist comparison with previous studies. Interneuronal correlations were quantified as trial-by-trial spike count correlations (rSC; also termed noise correlations), which were calculated for the two stimulus types separately. In brief, for each site we correlated the z-scored SU and MU activity for repeated trials of the same stimulus type (Fig. 1a). The resulting correlation coefficients (rSC) represent the common trial-by-trial fluctuations of shared ‘noise’ between the SU and surrounding MU in response to a given stimulus. The average rSC for all sites probed with the ambiguous cylinder stimulus was rSC = 0.3 (n = 88) (Supp. Fig. 1), rising to rSC = 0.37 (n = 52) for sites with matching and strong SU and MU tuning (see Methods). In contrast, rSC in response to the random motion stimulus was rSC = 0.16 (Supp. Fig. 1) on all sites probed with this stimulus (n = 53) and rSC = 0.17 on sites with matching and strong direction tuning (see Methods) (n = 41). The rSC values obtained for the random motion stimulus are in accordance with those found previously in V5/MT, analysing correlation for pairs of SUs recorded from a single electrode^36,50^.

Next, we directly compared rSC values for bistable cylinders and random motion for sites that were recorded with both stimuli pseudo-randomly interleaved (n = 52). rSC was consistently higher in response to the ambiguous cylinder than to random motion at the same site (Fig. 1b; p < 0.001, n = 52, *sign test*). This difference is also evident when comparing mean rSC for both stimuli (p = 0.002, n = 52; *t-test on Fisher’s z*) (Fig. 1c) and holds true for cells with matching and strong tuning (p = 0.023, n = 26) despite an increase in rSC values for both stimulus types. Data analysed separately for individual monkeys showed the same trend (Supp. Fig. 2).

Spike count correlations can depend on firing rate^52^. However, we found no significant correlation between rSC and mean firing rate (geometric mean of SU and MU firing rates), within either stimulus condition (ambiguous cylinder: r = 0.017, p = 0.9; random motion: r = −0.09, p = 0.53; *Pearson correlation*; n = 52 sites), nor was there any significant correlation between the change in rSC between the two stimuli and the difference in mean firing rate (r = −0.1; p = 0.46; *Pearson correlation*; n = 52 sites). Thus, the stimulus-related differences in rSC were not merely linked to changes in mean neuronal firing.

Previous studies observed a positive relationship between rSC and measures of tuning similarity in visual cortex^32^, specifically in area V5/MT^36,53,54^. Those observations suggest that shared common input endows nearby neurons with similar tuning properties and makes them subject to similar noise sources. We quantified tuning similarity using the Pearson correlation between SU and MU tuning curves (r_Signal_). For the coherently moving random dot stimulus, average r_Signal_ was large and positive: r_Signal_ = 0.68; and similar to that for ambiguous cylinders: r_Signal_ = 0.62. There was a weak relationship between rSC and r_Signal_ for the ambiguous cylinder (r = 0.25, p = 0.017, n = 88; *Spearman rank*). This observation was matched by a similar, non-significant trend for the random motion stimulus (r = 0.21, p = 0.16: *Spearman rank*) (Supp. Fig. 3a). Additionally, for pairs with matching tuning properties (see Methods) we find a positive correlation between rSC and the mean tuning strength i.e. mean F_cylinder_ of SU/MU pairs for the ambiguous cylinder (r = 0.32, p = 0.006, n = 71; *Spearman rank*). This effect remained significant when applying multiple linear regression (using z-transformed correlation values), factoring out firing rate (β = 0.48, p = 0.008). Again, for the random motion stimulus, a similar trend was observed (r = 0.24, p = 0.13, n = 41; *Spearman rank*) (Supp. Fig. 3b). Tuning strength for motion direction (F_motion_) was assessed on a coarse scale and therefore appeared larger than its equivalent for the cylinder rotation, for which F_cylinder_ was assessed with near-threshold disparities close to zero.

### Higher spike count correlations for the cylinders are linked to perceptual ambiguity

**Figure 2:**
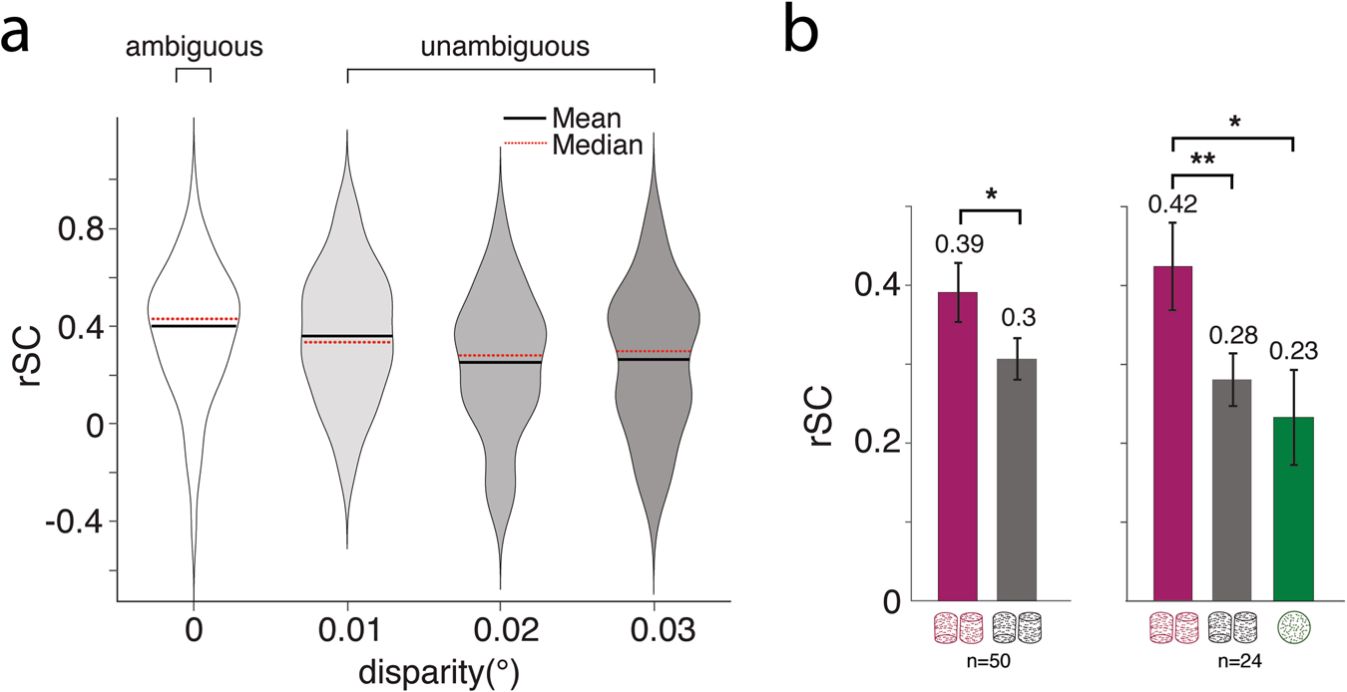
Spike count correlations for intermediate cylinder disparities. (**a**) Violin plots show distributions of rSCs for cylinder stimuli with different disparity strengths (p = 0.01; *one-way Anova on Fisher’s z*). Only disparity values tested on more than 10 recording sites were included; negative disparities were inverted so that each distribution is made up of rSC values obtained from both negative and positive cylinder disparity (i.e. both rotation directions). (**b**) Bar plots indicate mean rSC values for three stimulus conditions (purple: ambiguous cylinder; grey: unambiguous cylinder; green: random motion). Only sites with strong and matching MU and SU tuning (see Methods) were included. Left panel: Mean rSC values for all sites that were tested with the ambiguous cylinder (zero disparity) and at least one unambiguous cylinder stimulus (i.e. non-zero disparity). Right panel: Mean rSC values for sites that were tested with all three conditions i.e. stimuli. *p < 0.05; **p < 0.01; *t-test on Fisher’s z*. Error bars indicate s.e.m.

Ambiguous cylinder and random motion trials differed in both the visual stimulus and the task performed (perceptual choice for cylinders, fixation for random motion). But all recording sites were also probed with a number of non-zero disparity cylinder stimuli, which made it possible to compare rSC for different levels of stimulus ambiguity during performance of the same task. We observed that rSC decreased monotonically as the absolute value of disparity rendering the cylinder unambiguous increased (p = 0.01; *one-way Anova on Fisher’s z*) (Fig. 2a). We only included cylinder disparities that were probed for at least ten sites. Thus, the included disparities ranged from 0° to 0.03° in steps of 0.01°. On average, ambiguous cylinders evoked a larger rSC than unambiguous cylinders, when different non-zero disparity cylinder were pooled for each site (p = 0.02, n = 50, *paired t-test on Fisher’s z*) (Fig. 2b, left panel). The difference became even more pronounced when we limited our analysis to sites that were recorded with all three stimulus conditions (p = 0.006, n = 24, *t-test on Fisher’s z*) (Fig. 2b right panel). There was no significant difference between the average rSC for unambiguous cylinder and the random motion condition (p = 0.65, n = 24, *t-test on Fisher’s z*). Taken together, those results suggest that the level of perceptual ambiguity of the cylinder stimulus and not the decision task itself, is linked to high rSC. The results plotted in Fig. 2 apply for the case of the sub-set of neurons with strong and matching tuning but differences between stimuli were qualitatively similar when taking all recorded cells into the analysis. Data analysed separately for individual monkeys demonstrated the same trends (Supp. Fig. 4).

### Longer timescales of interneuronal correlations for ambiguous SFM cylinder

Our data show a higher rSC in response to ambiguous cylinders than to the unambiguous version of the same stimulus or to zero-coherence random dot motion. However, spike count correlations were measured using the average firing rate across the whole trial. In the following, we used the spike times from the same data to compare the cylinder and random motion stimuli in terms of spike time cross-correlations. This finer grained analysis allows to infer possible sources and structure of the correlation signal underlying the difference evoked by distinct stimuli.

**Figure 3:**
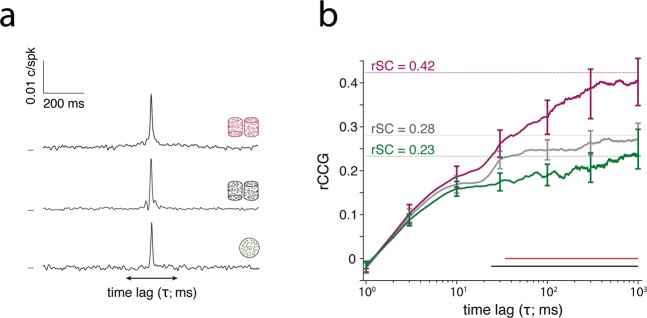
Spike time correlations and timescales of correlations. (**a**) Averaged CCGs for all SU/MU pairs recorded for the ambiguous cylinder (purple), the unambiguous cylinder (grey) and the random motion (green) stimuli. Only sites were included that showed matching and strong SU and MU tuning (see Methods) (n = 24). CCGs were smoothed with a Gaussian kernel (10 ms s.d.). (**b**) Average rCCG plotted as a function of the time-lag (τ), i.e. the integration window, for the ambiguous cylinder (purple), the unambiguous cylinder (grey) and the random motion (green) stimuli. Horizontal black and red lines indicate a significant difference between the purple and green and the purple and grey line, respectively (p < 0.01, *cluster based permutation test*, n = 24). Horizontal dashed lines indicate the corresponding average rSC values. rCCG courses start off at values below zero, because at time-lag zero there are no coincident spikes due isolating the SU from the MU activity on the same electrode. Error bars indicate the s.e.m.

We cross-correlated SU and MU neuronal spike trains on a trial-by-trial basis to obtain spike time cross-correlograms (CCGs) for each site and each stimulus condition, based on the method of Bair *et al*.^36^ . We compared the average CCG for sites with strong and matching tuning (n = 24) that were recorded under all three stimulus conditions (ambiguous cylinder, unambiguous cylinder and random motion) (Fig. 3a). The normalized area under the full CCG provides values mathematically identical to rSC^36^. Therefore, integrating the CCG over variable lags can reveal the timescale over which rSC arises. We plotted the integral under the CCG, termed rCCG, as a function of the time-lag (τ) (i.e. half width of the integration window centered at ~0) (Fig. 3b). At small integration windows τ < 20 ms, rCCG for all stimuli were similar. At longer time-lags (τ > 20–30 ms), rCCG values for the ambiguous cylinder kept increasing until around τ = 400 ms, becoming significantly larger than for both unambiguous cylinders and random motion (p < 0.01; *cluster-based permutation test*). rCCG time courses for the two latter stimuli appeared to plateau relatively early (τ ~ 10–30 ms) and were not significantly different from each other. Our observations for random motion stimuli correspond to those previously reported for SU-SU pairs, which found that rCCG for random motion reliably captured most of the rSC at a τ of 32 ms^36^. As expected, reported rCCG values approach corresponding rSC values for each condition (Fig. 3b).

These data suggest that while a substantial part of rSCs evoked by all three stimuli arises similarly on short timescales (around τ = 10 ms), a large part of the correlation associated with the ambiguous cylinder can only be explained by correlations on longer timescales (τ ~ tens to hundreds of milliseconds). These results therefore imply distinct sources of correlations that distinguish the processing of the different stimuli. Results were also replicated for each monkey separately (Supp. Fig. 5).

### Choice probabilities and timescales of correlations

Noise-correlations have been linked to perceptual choice signals measured as choice probability (CP) in a number of models^21,22,30,31,55,56^. Perceptually bistable SFM cylinder stimuli show relatively large CPs when compared to the random motion stimulus (0.67 vs 0.56)^8,10^. Therefore, we tested whether the size of the CP is associated with the magnitude or timescale of interneuronal correlations.

**Figure 4:**
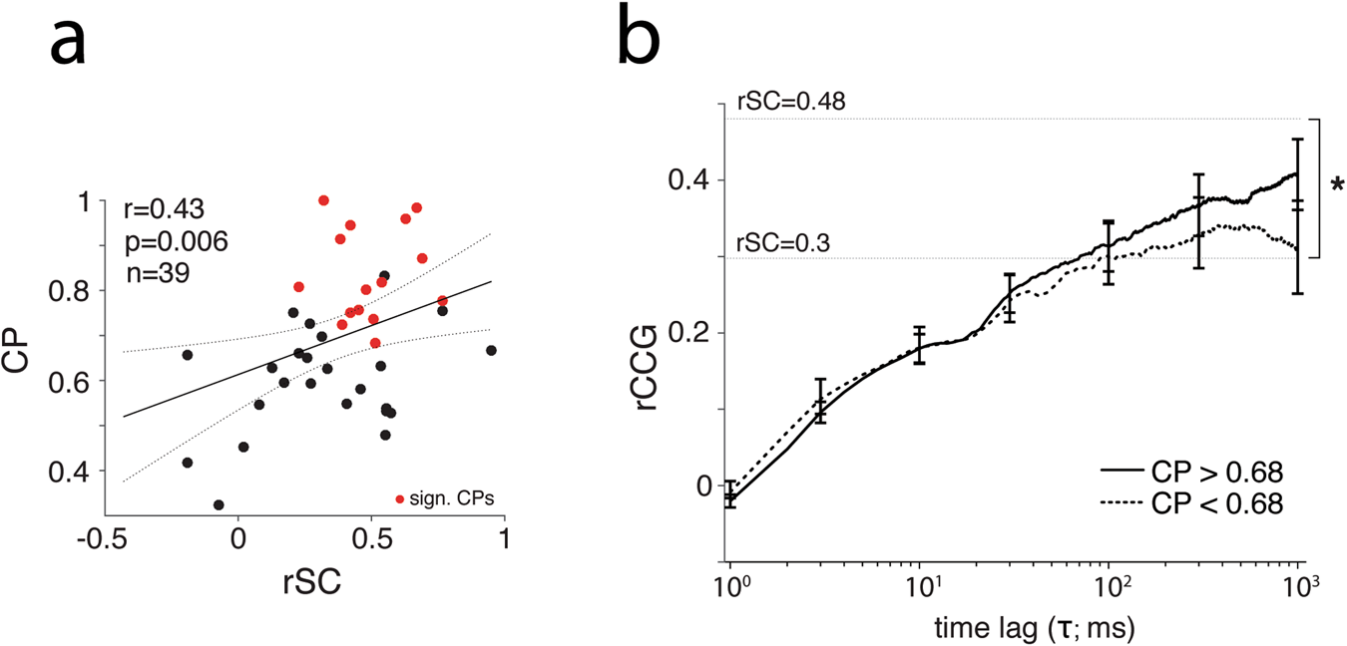
Choice probabilities and spike count correlations. (**a**) Scatter plot of SU CP and SU-MU rSC. Each dot represents a SU that showed strong and matching cylinder rotation tuning and for which a CP value could be measured (see Methods; n = 39). Red dots indicate SUs with a significant CP (p < 0.05; *cluster based permutation test*). Black solid line indicates a linear fit to all data points with 95% confidence interval (dashed lines) and displayed correlation coefficient and associated p-value stem from the Pearson correlation. (**b**) Average rCCG for subpopulations of sites split by the median CP associated with the SU (median CP = 0.68). Solid line: CP > 0.68; Dashed line: CP < 0.68. Error bars indicate the s.e.m. Horizontal lines indicate corresponding average rSC values. *p < 0.05; *un-paired t-test*.

We quantified the CP for the ambiguous cylinder stimulus and for the isolated SUs. We could not quantify CP for random motion presentations, since no choice was required of the monkeys. The average CP for all sites assigned a CP was 0.67 (n = 66; which was significantly different from chance (0.5), p < 0.01, *permutation test*), the same value found by Dodd *et al*.^10^, with which this data set overlaps. For sites with strong and matching SU-MU tuning for cylinder rotation the average CP was even higher: 0.69 (n = 39). Within this sample, neurons with high CPs for the cylinder were associated with higher spike count correlation values (r = 0.43; p = 0.006; n = 39; *Pearson’s r*) (Fig. 4a). As expected from previous assessments of neurometric thresholds^57^, we also observed a strong correlation between CP and tuning strength measured as F_cylinder_ (r = 0.65; p < 0.001; n = 39; *Pearson’s r*). Since rSC also depended on F_cylidner_ (r = 0.29, p = 0.05), any relationship between CP and rSC might therefore be confounded by the correlation of either with F_cylinder_. To control for this, we computed the partial correlation between CP and rSC, taking into account the effect of F_cylinder_ (r = 0.34; p = 0.03, *linear partial correlation*), which, although reduced, remains significant.

Next, to link CP to timescales of correlations, we split the population of neurons with strong and matching tuning (n = 39) by the median CP and computed the average rCCG courses for each half (Fig. 4b). Additionally, we computed the average rSC values for each half. As before, neurons with higher CPs (CP > 0.68) showed higher spike count correlations (0.48 vs. 0.3; p = 0.03; *un-paired t-test*). Differences between the rCCG time courses for each half appeared to emerge on relatively long timescales (τ > ~30 ms), but differences indicated a trend that was not significant. Taken together, high CP values associated with the ambiguous cylinder might be explained by interneuronal correlations acting on longer timescales. Trends were similar when data were analysed for each animal separately (Supp. Fig. 6).

### Interneuronal correlation time-course predicts choice probability

So far, the measures of interneuronal correlation (rSCs, rCCGs) used here do not distinguish processes happening at specific time-points during the trial. A recent modelling study^21^ using the random motion stimulus found that correlations induced by sensory input arose relatively early in the trial and stayed constant during the trial, showing only weak time-lagged components. In contrast, correlations due to a top-down feedback component rose throughout the trial and showed a strong component at longer time-lags. Furthermore, time-courses of lagged top-down induced correlations were predictive of CP signals^21^. Here, we characterized interneuronal correlations arising at different time-points and timescales during the trial for all stimulus conditions and show how those correlations can predict CP trajectories for the ambiguous cylinder.

**Figure 5:**
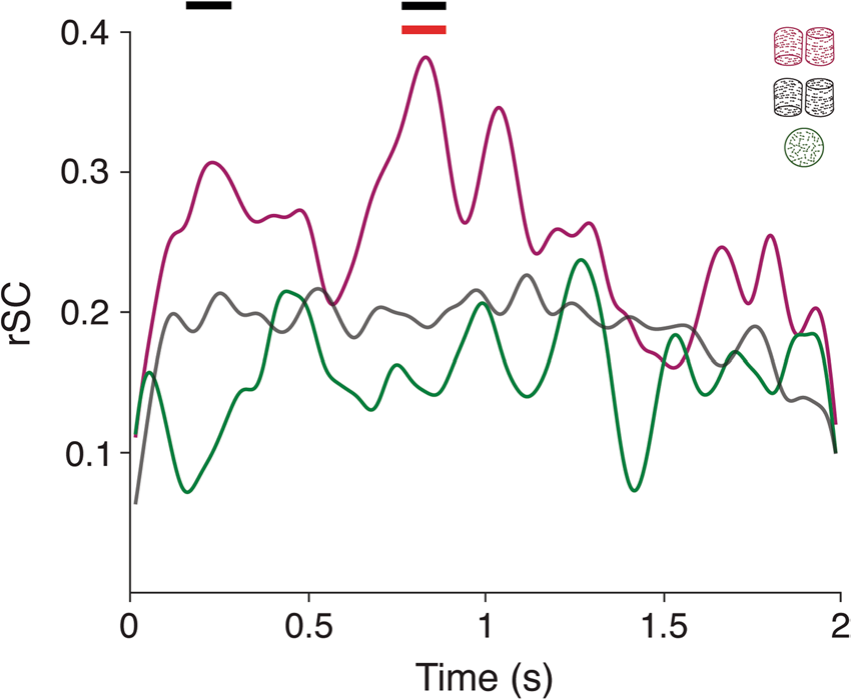
Time-course of spike count correlations. Average rSC time-courses estimated from a sliding 100 ms window. Purple, grey and green lines show the average rSC time-courses for the ambiguous cylinder, the unambiguous cylinder and random motion, respectively. Averages were computed over all sites recorded under all three stimulus conditions (n = 24). The black horizontal bar indicates a time window with a significant difference between the ambiguous cylinder and the random motion condition (p < 0.01; *cluster based permutation test*). The red horizontal bar indicates a significant difference between ambiguous and unambiguous cylinder conditions (p = 0.01). Time-courses were smoothed with a 30 ms (s.d.) Gaussian kernel for display. All statistics were performed on the un-smoothed traces.

First, we computed rSC time-courses using a 100 ms sliding window averaged over sites that were recorded under all three stimulus conditions and that showed strong and matching tuning (n = 24) (Fig. 5). This instantaneous rSC for the ambiguous cylinder ramps up relatively early in the trial, when it is significantly higher than for the random motion stimulus (p = 0.01, *cluster-based permutation test*). It is highest towards the middle of the trial (~800 ms) [significantly higher than for both random motion (p = 0.001) and unambiguous cylinder (p = 0.01) stimuli]. During the second half of the trial, instantaneous rSC values are similar in magnitude for all three stimulus conditions. Those results indicate that the higher correlations setting apart the ambiguous cylinder arises during the first half of the trial. Similar results were obtained for each monkey separately (Supp. Fig. 7).

**Figure 6:**
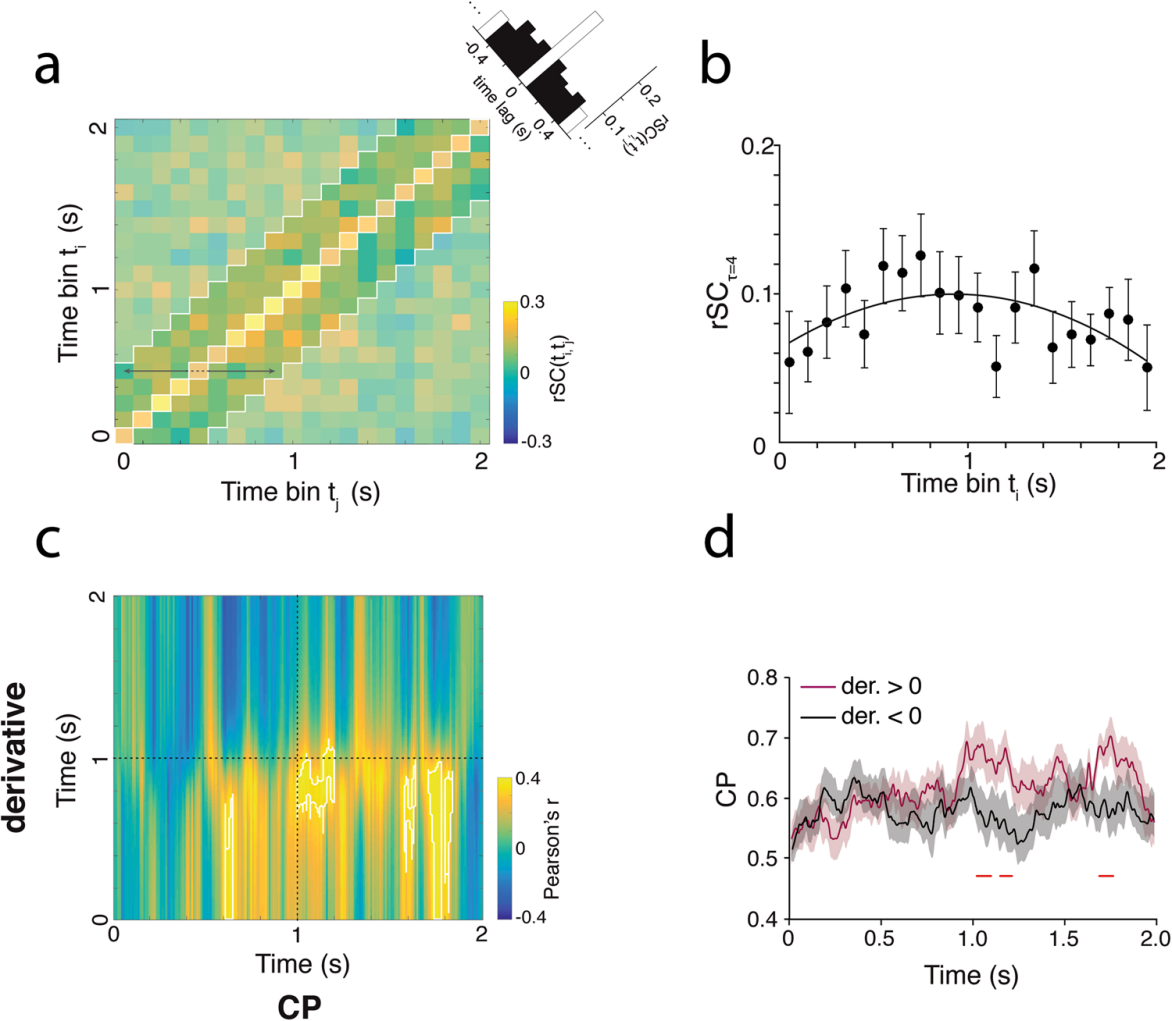
Correlation time-course predicts choice probability. (**a**) The spike count correlation matrix for the ambiguous cylinder: The matrix entries rSC(t_i_, t_j_) are the rSC values computed from time-bins t_i_ in the SU and t_j_ in the MU (100 ms independent time-windows) and averaged over all sites. The color bar indicates the magnitude of the rSC values. The more saturated colours indicate off-diagonal, i.e. the four lagging and leading time-bin pairs with respect to t_i_ (excluding the main diagonal; see black arrows), that were used to compute rSC_τ=4_. Inset shows histogram of the rSC values averaged along the main diagonal. Black bars show the diagonals that were used to compute rSC_τ=4_ (**b**) rSC_τ=4_ averaged over all sites are plotted as a function of time-bins t_i_. Error bars show s.e.m. The black line indicates a 2^nd^ order polynomial fit to the rSC_τ=4_. (**c)** The matrix for Pearson correlation coefficients was computed between the derivative of the fitted polynomial in b) and CP across recording sites. Matrix entries simply represent the correlation coefficients obtained for each possible time-point pairing. White contours mark clusters with a significant Pearson correlation (p = 0.01; *cluster based permutation test*). The color bar indicates the magnitude of the correlation. Dashed black lines mark 1 s (i.e. half of the trial). (**d**) The CP time-courses averaged across sites with a negative derivative (black; n = 21) and positive derivative (purple; n = 18). The red horizontal lines mark significant differences between the two CP time-courses (p < 0.05; *cluster based permutation test*). Shaded area indicates s.e.m.

Next, we extended our analysis to incorporate correlations occurring at distinct timescales (i.e. different τ’s; as in Fig. 4) while preserving some of the time-resolved trial structure. Hence, for each site, we extended the analysis of Fig. 5 across different time lags. Specifically, we computed rSC for all pairwise combinations of independent 100 ms time-bins within the trial (t_i_ in SU and t_j+x_ in MU). We included sites associated with a CP and with strong and matching tuning for the ambiguous cylinder (n = 39). In the resulting rSC matrix (Fig. 6a), the central diagonal of the matrix is a down-sampled version of the rSC time-course in Fig. 5 and corresponds to correlations occurring at short timescales (τ < 50 ms), which we term instantaneous correlations. Data on the off-diagonals correspond to correlations at longer timescales. Averaging the rSC matrix across the main diagonal corresponds to a down-sampled version of the CCGs in Fig. 3a (insets in Fig. 6a and Supp. Fig. 8a).

To probe whether interneuronal correlations at longer timescales could be predictive of CP measured at subsequent points during the trial^21^, we isolated the long timescale component of the rSC from the rSC matrix. For each time-bin t_i_ we averaged over the four leading and four lagging rSC values (i.e. matrix entries) in respect to t_i_ (see black arrows in Fig. 6a) while excluding the on-diagonal rSC value. This average value is termed rSC_τ=4_ and represents the trial time resolved long timescale component of the rSC, corresponding to a lag of ±50–450 ms (Fig. 6b). The average rSC_τ=4_ time-course, although noisy, is qualitatively similar to the instantaneous rSC time-course in Fig. 5 and shows a strong rise up until ~800 ms, as well as a decay during the second half of the trial. In order to quantify these trends for each site, we fitted a 2^nd^ order polynomial to the rSC_τ=4_; the derivatives of the polynomial provide a trial-time resolved index of whether rSC_τ=4_ is rising, decaying or constant. Next, for each recording site’s SU, we computed CP time-courses in sliding 100 ms window. Thus, for each time-point (ms) in the trial, we have two vectors: one vector of the derivatives indicating an rSC_τ=4_ trend for each site and a second vector containing the CP values for each site. When we computed the Pearson correlation between the derivative and CP vectors for each pairwise combinations of time-points, the resulting correlation matrix revealed that a rising rSC_τ=4_ during the first half of the trial is associated with a larger CP values during the second half of the trial (p = 0.01; *cluster-based permutation test*) (Fig. 6c). In other words, the rSC_τ=4_ slope during the first half of the trial is predictive of CP magnitude later in the trial. This relationship is further illustrated when we split recording sites by the sign of their time averaged derivative i.e. whether their rSC_τ=4_, on average, rose or decayed throughout the trial. For each group (n = 21 for sites with derivative <0; n = 18 for sites with derivative >0) we averaged the CP time-courses (Fig. 6d). Similar to Fig. 6c,d shows that a rising rSC_τ=4_ is associated with higher CP during the second half of the trial (p < 0.05; *cluster-based permutation test*). Our data showed similar trends when analysed for each monkey separately (Supp. Fig. 8).

We then tested whether we could observe a similar relationship when taking into account only the instantaneous correlation, i.e. the short timescale component of the rSC (τ < 50 ms; denoted rSC_τ=0_). However, there was no clear relationship between rSC_τ=0_ and CP (Supp. Fig. 9). More specifically, the derivative of the polynomial fitted to rSC_τ=0_ did not predict CP at any time-point in the trial. Together, those results show that CP for the ambiguous cylinder is best predicted by the dynamics of correlations at long but not short timescales, which in turn might reflect a feedback signal onto V5/MT.

## Discussion

Our data show a significant difference between the interneuronal correlations evoked by distinct visual stimuli and tasks: Interneuronal correlations measured while animals made perceptual choices about the rotation direction of an ambiguous cylinder were higher (rSC = 0.42) than when the cylinder was rendered unambiguous (rSC = 0.28) or when monkeys maintained fixation while viewing a classic random dot stimulus (rSC = 0.23). Interestingly, the higher correlations for the ambiguous cylinder arose on longer timescales (100–400 ms), indicative of a top-down influence on sensory processing. Furthermore, the long timescale, but not the instantaneous component, of the interneuronal correlations was predictive of CP time-course associated with the ambiguous cylinder. These data present a potential link between a top-down source injecting interneuronal correlations into sensory areas of visual cortex and the choice signals measured therein.

The reported magnitude of measured spike count correlations in visual cortex is usually small and positive but can vary across brain region, stimulus or task instruction^32^. In V5/MT, reported values for rSC range from 0.1 to 0.2 for neural responses to random dot motion stimuli^28,36,50,53,54^. We compared the isolated single unit to the surrounding multi-unit activity, rather than comparing pairs of isolated SUs with the same electrode. Nonetheless, our measured rSC for the random motion condition (0.17) was very similar to the one measured by Bair *et al*.^36^ (0.19) and Zohary *et al*.^50^. In the present study, as well as the above mentioned studies, single cells were carefully selected for their responsiveness and tuning profiles to the main visual stimulus as well as tuning similarity, properties which positively correlate with rSC, generally^32^, and specifically in V5/MT^28,36,53,54^. Poor spike sorting can lead to inflated rSC values^40,46^, as can pooling of cells^58^. However, our measure of rSC for random motion also matches the rSC measured by Cohen and Newsome^28^, using the same stimulus, but recording isolated cell pairs with separate electrodes (rSC ≈ 0.2 for pairs with similar tuning).

Our data revealed a comparatively large value of rSC (0.37) in response to the ambiguous cylinder. In contrast to the random motion task, which purely required fixation, the cylinder task required a perceptual decision. While we could not directly compare decision-making conditions for both stimuli/tasks, our observations present evidence that it is stimulus-related properties rather than task instructions that determine rSC. For one, other data show that rSC measured for random motion does not differ markedly across task difficulties, i.e. the strength of motion coherence^36^ and passive fixation^50^. Furthermore, we found that the large rSC associated with the ambiguous version of the cylinder stimulus diminished when the cylinder stimulus was rendered unambiguous, approaching the value for random motion. Thus, the perceptual bistability of the cylinder stimulus and not the decision-making component seems fundamental to the generation of high rSCs.

The cylinder and random motion stimuli differ markedly in their perceptual interpretation. The random motion stimulus contains dots, which probabilistically move in all possible directions, resulting in zero net motion. This type of stimulus does not evoke a stable percept. In contrast, the ambiguous cylinder stimulus evokes two different percepts, each of which is stable: perceptual reports of the rotation direction indicate that there can be spontaneous switches between each of these two stable representations at a rate of tens of seconds^59,60^. Crucially, the dots forming the cylinder are consumed into a single perceptual representation in a ‘winner-take-all’ fashion, although the sensory input remains indeterminate in nature.

Human fMRI studies in bistable perception have suggested a fronto-parietal top-down component stabilizing/destabilizing the percept generated in sensory areas through a feedback mechanism^61–63^, which might affect the circuits in V5/MT. Some evidence supporting this interpretation in our task comes from the longer reaction times (RTs) that are displayed by monkeys when deciding on the rotation direction of the ambiguous cylinder as opposed to the unambiguous version. This could be interpreted as showing that the signals stabilizing the percept become more dominant when the sensory input is weak or absent (Cicmil *et al*., personal communication). Alternatively, transitions in bistable vision could be a result of dynamic adaptation and inhibition within visual cortex. These processes do not necessarily rely on a feedback modulation but could arise in a feed-forward fashion^64^ or through attractor mechanisms^65,66^, which might also influence interneuronal correlations^67,68^.

Correlations in the activity of sensory neurons arise over a variety of timescales^32,34,36^. We show that much of the correlation structure that is specific to the ambiguous cylinder arises on relatively long timescales (tens to hundreds of milliseconds). In contrast, correlations associated with the random motion stimulus saturated at a time-lag of about 30–40 ms, which matches a previous study by Bair *et al*.^36^. This was also the case for unambiguous versions of the cylinder stimulus. Experimental and theoretical studies have shown that feedback signals onto sensory cortex that represent cognitive factors such as attention, learning or internal bias, affect the structure of noise correlations therein^21,24,28,30,43,44,56,69–71^. The timescale of fluctuations of such top-down cognitive factors is estimated to be comparably long. For example, fluctuations in attention range from hundreds of milliseconds to seconds^72,73^, but not less than 100–200 ms^47^.

To determine the signature of different sources of correlations, Wimmer *et al*.^21^ explored a hierarchical network model designed and trained to solve a direction discrimination task with random motion stimuli. Their model consisted of a sensory layer (representing V5/MT) which was recurrently connected to a higher order integration layer (representing LIP or FEF). It made it possible to disambiguate the correlation signatures in the sensory layer that were injected by the feedback signal (termed top-down correlations) versus those accounted for by the local fluctuation in the sensory layer and the stimulus (termed bottom-up correlations). Their modelling found that bottom-up correlations depended on interactions at short time-lags and disappeared at longer time-lags (>125 ms). Conversely, top-down correlations were weak for short time-lags but stronger for longer time-lags (>125 ms), especially during the second half of the trial. Interestingly, they were overall negligible at the beginning of the trial but rose towards the end. The structure of noise correlations observed in that model matched those of real V5/MT neurons from monkeys performing the motion discrimination task^21^. In short, lagged correlations rose throughout the trial while instantaneous correlations were flat.

In our data, however, the correlation time course for the ambiguous cylinder showed a prominent bump in the middle of the trial. Like in Wimmer *et al*. 2014, our correlation time-course at longer time-lags showed a rising phase but then peaked in the middle, unlike in Wimmer *et al*. where the lagged correlation time-course continued to rise until the end of the trial. Importantly, a great deal of the interpretation offered by that model is valid only under the assumption that the animal takes the decision about how to respond at the end of the trial. Some evidence has been put forward that this might be the case using different paradigms^74^. Furthermore, the random motion stimuli used in the model are noisy, do not evoke a consistent percept of motion direction and require prolonged integration of evidence. In contrast, the ambiguous version of the cylinder stimulus might evoke a clear ‘winner take all’ percept earlier in the trial.

If there were a feedback signal biasing perception towards one of the two possible configurations, this could act relatively early in the trial and might explain the observed bump in the rSC timecourse for the ambiguous cylinder stimulus (Fig. 6). Intriguingly, this bump is not present in the instantaneous rSC timecourse for the unambiguous cylinder or random motion stimuli, which seem more similar to the flat instantaneous time-courses observed in Wimmer *et al*. 2014^21^. A similar flat time course was also observed in a more recent study by Bondy *et al*.^24^, who used unambiguous and noisy stimuli unlike the ambiguous cylinder. Additionally, the observed rise and decay in correlations causing the distinct bump might mark the engagement and disengagement of attentional processes quenching the correlations in V5/MT^43,69,75^, which could act from the beginning of the trial^49^.

More generally, a top-down component dynamically changing correlations between relevant neuronal pools^24,28^ is also in accordance with pooling models for the cylinder discrimination task^20,29,57^, that suggest that neuronal pools must be structured flexibly and independently for successful discrimination of stimuli. We note that feedback onto V5/MT need not be of a general ‘cognitive nature’. The cylinder stimulus requires the specific conjoint read-out of motion and disparity cues^7^. The medial superior temporal area (area MST), which lies upstream of V5/MT, shows disparity-dependent direction selectivity potentially related to the perception of rotation^76^. Area MST might send feedback signals related to the perception of rotation to V5/MT increasing the correlation between groups of neurons signalling the same rotation.

Here, our experimental results show a positive correlation between the rSC of a single unit with its surrounding multi-unit activity and that single unit’s CP. Previously, theoretical models have proposed a relationship between the rSC of a given neuron and its CP^22^. Haefner *et al*.^30^ derived analytically that a neuron’s CP should be determined by the mean correlation between that neuron and all other neurons, weighted according to a pooling weight for each neuron. The direct correlation we find between CP and rSC might be related to our estimation of rSC, the selection criteria and sampling of our recorded neurons: Our calculation of rSC is not pair-wise but for a given neuron with a number of its direct neighbours, probably situated in a column with similar tuning properties. In general, SU and MU showed very similar tuning (we only found 4 neuronal sites showing a clear tuning mismatch). Also, our selection criteria required a minimum for stimulus selectivity for both single and multiunit. Thus, our sampling/selection would prefer similarly tuned, spatially close neurons with presumably ‘higher weights’ because of their task relevance. However, the columnar architecture in V5/MT means that correlations between neural signals that are spatially very close to each other are a theoretically important part of the population’s overall correlation structure. Nevertheless, a limitation of the current study is the small sample size and recording technique which prevent us from estimating the full correlation matrix, necessary to validate the exact predictions made by Haefner *et al*.^30^.

A recent study recording from multi-electrode arrays in V1 in the macaque monkey did provide this missing link and predicted CP accordingly^24^. Interestingly, they also found that the largest variability in the rSC structure seemed to be determined by a task-dependent feedback component, instead of a purely feed-forward component considered in Haefner *et al*.^30^. This observation and the direct rSC/CP correlation in our data may have a similar explanation. The correlation could be driven by a dynamic feedback signal commonly affecting the measured rSC and CP, which could also explain our observed positive correlation between rSC and CP after taking away the effect of stimulus tuning. Supporting this notion, we also find some evidence that CP is better predicted by the long timescale component of rSC. More specifically, a rising lagged component of the rSC during the first half of the trial predicted larger CPs during the second half of the trial. To some extent, this is in accordance with previous results for the random motion discrimination by Wimmer *et al*. 2014^21^, mentioned previously, where rising lagged correlations (possibly representing top-down feedback) correlated with larger CPs towards the end of the trial^21^. In our, case lagged correlations decayed towards the second half of the trial while CPs remained constant. This might indicate differences in decision or attentional timescales as mentioned before. In short, a decision about the ambiguous cylinder might be conveyed during the first part of the trial, without prolonged need for a top-down feedback towards the end of the trial. Of course, we cannot fully determine the source of the long timescale correlations without simultaneous recordings in other brain areas. But our observations seem in accordance with a rising body of experimental^16,24,27^ and theoretical studies^55,56,77^ suggesting a top-down component conveying decision-related signals in sensory areas and specifically V5/MT^20,42,47,66,78^.

Lastly, a recent study re-evaluating common measures of CP during 2AFC tasks in ventral parietal area (VIP) found that CP magnitude can be overestimated when computing stimulus tuning curves from trials collected during the actual decision-making experiment^79^. This is because part of CP is decoupled from sensory input and rather reflects a purely choice dependent top-down signal that distorts the ‘true’ sensory tuning curves, which are then used to compute a possibly inflated CP. However, the tuning to cylinder disparity in visual area V5/MT is independent of task performance and the magnitude of CP for the cylinder stimulus is correlated with neurometric threshold^10,80^. Together with micro-stimulation experiments^7^, it is clear that V5/MT neurons contribute directly to perceptual decisions about SFM cylinders.

In summary, we show that bistable SFM cylinders associated with significant choice-related activity (CP), evoke highly correlated neuronal firing (rSC) between nearby neurons in visual area V5/MT. Much of the interneuronal correlation setting apart the bistable cylinder percept from unambiguous versions as well as classic random motion stimulus arises on relatively long timescales. It is these correlations on longer timescales that predict the particularly large choice-related component of neuronal activity observed for the bistable cylinder. Here, we argue that the relationship between interneuronal correlations and choice-related firing might be the neural signature of a feedback signal acting upon V5/MT circuits, stabilizing the perceptual appearance of the SFM cylinder. Bistable, ambiguous stimuli like the SFM provide an interesting probe for models of perceptual decision-making, in particular to investigate differential contributions of bottom-up and top-down influences during perceptual decisions.

## Methods

Physiological recordings have been reported in detail before^10,81^. In brief, two adult rhesus monkeys (*Macaca mulatta*) were trained to perform a two alternative forced choice task (2AFC) for fluid rewards. A headpost, scleral search coils and a recording chamber were implanted. We recorded a total of 93 sites (29 in monkey bill and 64 in monkey rin) in extrastriate visual area V5/MT using single microelectrodes. Stimuli and perceptual tasks were optimised for the disparity and motion tuning of each recorded single neuron. All procedures were performed in accordance with United Kingdom Home Office regulations and European Union guidelines on animal experimentation (EU directive 86/609/EEC; EU Directive 2010/63/EU). Animal protocols passed local and national ethical review and were licensed by the UK Home Office.

### Stimuli and experimental paradigm

Cylinder stimuli are defined by two transparent planes of randomly placed (seeded anew every trial – i.e. not frozen noise) black and white dots moving in opposite directions on a mid-gray background; for further details of the stimuli, set-up and task see^10^. The velocity of the dots is a sinusoidal function of spatial position, which gives rise to a sensation of depth-from-motion. When no depth order is specified (zero binocular disparity), the direction of rotation of the cylinder is ambiguous. For long durations of viewing (several seconds or more), the stimulus is perceptually bistable and spontaneously flips its perceived configuration^59^. For shorter durations (including the 2 s presentations used here), the stimulus is stable but changes from one presentation to the next^10,60^. Adding binocular disparity to the moving dots provides sensory evidence that disambiguates the motion percept into rotating clockwise (CW) or counterclockwise (CCW). The amplitude of the disparity signal controls the degree of perceptual ambiguity of the cylinder. As the amplitude of the disparity signal increases, the variability of the judgments decreases and the reported motion rotations resolve into unambiguous decisions in favour of CW or CCW rotation.

The ambiguous and unambiguous cylinder stimuli were presented in a pseudo-random sequence of trials, into which presentations of random motion stimuli were also pseudo-randomly interleaved. These stimuli had a binocular disparity of zero and their dots had a lifetime of one frame. At the beginning of each trial the monkeys were required to fixate on a point in the middle of the screen. Upon successful fixation, a stimulus was presented in the receptive field of the recorded neuron for 2 s. On cylinder trials, two targets appeared on either side of the fixation marker after stimulus presentation and the monkey had to indicate its perceptual choice about rotation direction with a saccade. Animals received a fluid reward for maintaining fixation and making a correct choice about the rotation direction of the unambiguous cylinder. Reward was given at random for the ambiguous cylinder. In response to presentations of random motion stimuli, animals were rewarded for maintenance of fixation only, no choice targets appeared and no choice was required.

### Spike sorting

We used Parylene-coated tungsten electrodes with an impedance of around 0.5–1.2 MOhm (Microprobe Inc.) that allowed clear single unit (SU) recordings. After amplification (BAK electronics), filtering (200 Hz to 5 kHz) and digitization (32 kHz), electrode signals were stored to disc as well as classified online (DataWave Technologies). Online, experiments were only carried out when an SU could be clearly separated from the lower amplitude multi-unit (MU) activity and the SU had clear direction and disparity tuning, to which the test stimuli were matched (for details of this experimental procedure see Dodd *et al*.^10^).

Spike sorting was repeated *de novo* on the stored electrode signals offline in a custom-built C-programme. For each recording site to be included, the SU activity had to have a clearly distinguishable spike waveform that was visually checked and maintained throughout all the experiment runs. Quantitative analysis of the spike waveforms was performed using a range of parameters, most commonly ‘spike width’, ‘spike energy’, ‘spike excursion’. In order to be included, the SU spike values had to form a separate cluster away from the remaining MU activity – for all measures. The selection for the SU was adjusted until only the spikes forming this cluster were included. The MU activity was sampled by taking signals above a Schmitt Trigger level adjusted for each experiment minus the identified SU activity, so that the MU activity for 0° disparity cylinders was between 10 s to 200 spikes/s.

There inevitably is a small fraction of spikes that are misclassified in any computational sorting method. To test whether our results were robust to small errors in spike classifications, we simulated deliberate misclassifications. In short, we assigned varying fractions of random spikes from the SU to the MU and vice versa to create shuffled spike trains. We repeated the analysis of our main results (Fig. 3) using the shuffled spike trains to confirm that our results were not explained by moderate errors in spike sorting.

### Data analysis

All analysis was performed on spike sorted data. For all correlation analysis we compared the isolated SU with the exclusive MU response recorded on the same electrode. We excluded sites with a firing rate <10 spikes/s estimated across trials and excluded trials containing less than one spike. Furthermore, we required activity to be recorded for at least five trials for a given condition. Those criteria eliminated 5 sites from our original sample of 93 sites (n = 88). Included sites showed significant SU tuning for binocular disparity over a wider range (±0.9°) and direction of motion (360°) (p < 0.05, *ANOVA*). Analyses were implemented using custom code written in Matlab (The MathWorks Inc., Natick, MA).

#### Estimation of tuning parameters

Fitting of tuning curves and parameter estimation was performed on the square root of the firing rate (spikes/s) for each 2 s stimulus presentation to remove the linear relationship between variance and firing rate^82,83^. Curve fitting was performed using the Matlab implementation of Nelder-Mead simplex algorithm^84^, which sought a local minimum to the squared error. All procedures were carried out for SU and MU activity, if not indicated otherwise.

To estimate motion direction tuning, recording sites were probed with a patch of dots coherently moving across the receptive field at a minimum of 6 different directions, over a range of 360°. Mean responses to motion directions were fit with a von Mises distribution. Von Mises distributions generally provided excellent fits to the data, accounting for >90% (median across neurons) of the variance in the mean response across directions. Tuning for the direction of cylinder rotation (CW vs CCW) was estimated in responses to cylinder stimuli presented over a narrow range of cylinder disparities around 0°: −0.06° to 0.06°. Here, negative disparities indicate CW rotation whereas positive disparities indicate CCW rotation. A cumulative Gaussian was fitted to the mean response for each cylinder disparity, applying the same procedure as for the direction of motion tuning. Cumulative Gaussians provided a good fit accounting for >90% (median across neurons) of the variance in the mean response across cylinder disparities.

To extract parameters describing the strength of selectivity to a given parameter (motion direction or cylinder rotation), we calculated the F-index^82^, given by

1 $${F}_{index}=\frac{M{S}_{treatment}}{M{S}_{error}+M{S}_{treatment}}$$

where, MS_*error*_ and MS_*treatment*_ represent the familiar mean square terms from a one-way ANOVA performed on the trialwise responses to either cylinder stimuli or moving random dot stimuli. MSE_*error*_ is the mean within disparity or motion direction variance, and MS_*treatment*_ is the mean between disparity or motion direction variance. The F_*index*_ quantifies the modulation of the tuning function by the tested parameters (neuronal property) while taking into account the reliability of the measured responses (experimental property)^82^. We calculated F_*indices*_ for both cylinder rotation (F_*cylinder*_) and motion direction tuning (F_*motion*_), respectively. Tuning of MU and SU was deemed as strong if F_index_ was greater than 0.1.

Tuning similarity between SU and MU responses were evaluated using the r_*Signal*_ metric, which refers to the Pearson correlation between two raw tuning functions. Additionally, for cylinder rotation tuning, we assessed similarity by comparing the maxima of the two cumulative Gaussians fits for SU and MU, which had to lie within the same rotation direction (CW vs CCW) as indicated by the sign of the disparity. For motion direction, the maxima of the von Mises fits had to lie within 90° of each other to be considered as similarly tuned.

#### Spike count correlations

Spike count correlations (rSC) (also known as noise correlations)^32,50^ were calculated using repeated trials within a condition. Spikes were counted for each trial over the full 2 s duration and converted to spikes/s. To avoid contamination of our estimates of r_sc_ by outlier responses, we removed trials for which the response of either SU or MU was >3 SDs different from its mean response computed over all trials^50^. We then normalized firing rates on each trial for both mean firing and slow drifts in neural excitation by computing a z-score for SU and MU firing rate on each trial using a sliding window of 5 trials before and after the current trial^28^. The z score for the *i*^*th*^ neuron (=SU or MU) on trial *k* is defined as:

2 $${z}_{i}(k)=\frac{{r}_{i}(k)-{\mu }_{i}}{{\sigma }_{i}}$$

in which, *r* is the firing rate on trial *k* and *μ* and *σ* are the mean and standard deviation of the *i*^*th*^ neuron firing rate on the previous and following 5 trials. We then computed rSC as the Pearson correlation between the SU and MU z-scores. All statistics involving rSC values were carried out after transforming them to Fisher’s z.

#### Spike time correlations

We computed spike train cross-correlograms (CCGs) and auto-correlograms (ACGs)^85^ using binary spike trains at a 1 ms resolution. The trial-averaged cross correlation of two spike trains is defined as:

3 $${C}_{jk}(\tau )=\frac{1}{M}\mathop{\sum }\limits_{i=1}^{M}\,\mathop{\sum }\limits_{t=1}^{T}\,{x}_{j}^{i}(t){x}_{k}^{i}(t+\tau )$$

where *x*_*j*_^*i*^ or *x*_*k*_^*i*^ can be 1 or 0 depending whether neuron *j* or *k* (=MU and SU) fire an action potential at time-point *t* in trial *i*. *T* is the duration of the trial in milliseconds and *M* the number of trials. The parameter *τ* defines the time-lag (in ms) i.e. the time-window the cross-correlation is computed over. For the auto-correlation, *j* = *k*. To compute the CCGs and ACGs, the trial averaged cross-correlation is simply divided by the product of a triangular function and the geometrical mean of the mean firing rates of neuron *j* (*λ*_*j*_) (e.g. SU) and *k* (*λ*_*k*_) (e.g. MU):

4 $$CCG(\tau )=\frac{{C}_{jk}(\tau )}{\Theta (\tau )\sqrt{{\lambda }_{j}\ast {\lambda }_{k}}}$$

The triangular function *Θ* (*τ*) represents the extent of overlap of the spike trains as a function of the discrete time-lag *τ*:

5 $${\rm{\Theta }}(\tau )=\,T-|\tau |,\,(\,-\,T < \tau  < T)$$

i.e., that there are *T* opportunities for simultaneous events in a trial of length *T* but only *T*-1 opportunities for coincidences at time-lags of 1 ms, etc. Substituting equation (5) into equation (4) corrects for the triangular shape of the C_*jk*_ caused by the finite duration of the data. We chose to normalized the CCGs and ACGs by the geometric mean spike rate, as opposed to the product of spike rates or another factor, for two reasons. First, it is the most commonly used normalization, so it facilitates comparison with previous studies^36,40,53,86–88^ [similar to the normalization of the joint peristimulus time histogram]^89^. Second, this normalization provides results that are most comparable with measures of spike count correlation^36,40^. In order to correct for stimulus induced co-variations in SU & MU firing rates, we computed a shift predictor:

6 $${C}_{jk}^{\ast }(\tau )=\frac{M{S}_{jk}(\tau )-{C}_{jk}(\tau )}{M-1}$$

in which S_*jk*_ denotes the cross correlation of the post-stimulus time histograms (PSTH)^36^. Substituting C_jk_^*^ for C_*jk*_ in equation (4) gives the final shift predictor, which was then subtracted from the CCGs and ACGs, respectively. We also computed CCGs, ACGs and shift predictors after excluding the first 300 ms of each trial to avoid processing the initial transient onset response, which could influence correlation strength. However, we found only negligible differences.

To calculate the timescale of correlations we used the rCCG metric derived by Bair *et al*.^36^.

7 $$rCCG(\tau )=\frac{{\int }_{\tau =-t}^{t}\,CCG(\tau )}{\sqrt{({\int }_{\tau =-t}^{t}\,AC{G}_{j}(\tau ))({\int }_{\tau =-t}^{t}\,AC{G}_{k}(\tau ))}}$$

The rSC is equal to the integral over the full shift predictor corrected CCG i.e. time-lags covering the full duration of the trial, divided by the geometrical mean of the respective integrals of the ACGs (also corrected by the shift predictor) of the MU and SU^36^. Integrating different areas around the central peak of the rCCG allows for an assessment of different timescales of correlations.

#### Choice probabilities

Choice probabilities (CPs) were calculated on the mean SU firing rate in response to the ambiguous cylinder over the stimulus duration (2 s). Trials were pooled according to the choice made by the monkey (CW vs CCW). CP is inferred by quantifying the separation between the two distributions. Specifically, this is done by calculating the area under the ROC curve^8,90^. We included neuronal data only from blocks of trials in which the animal’s behavioural performance demonstrated that it was accurately responding to the cylinder disparity. Therefore, we required that the animal scored over 80% correct at the largest values of cylinder disparity tested, typically 0.03°, and that the animal’s responses to the ambiguous cylinder were unbiased (experiments in which the same target was selected on more than 80% of the ambiguous cylinder trials were discarded). Our selection criteria led to the exclusion of 22 recording sites from our initial sample (n = 66).

#### Statistical testing using cluster-based permutation tests

Throughout this study we used a non-parametric cluster based permutation test^91^. The method compares some observed test statistic with a constructed null distribution while controlling for multiple comparisons across time. Depending on the question at hand we constructed null distributions in three ways: (1) By randomly permuting condition labels when comparing cylinder conditions with each other or with the random motion condition. (2) By randomly permuting CP values across sites when comparing median splits of the population or computing correlation coefficients across the population. (3) By randomly permuting correct and incorrect trials when evaluating CPs. The relevant test statistic or raw difference was computed for the observed data as well as for each of 10000 permutations. For (1) and (2), a two-sample t-test was applied for each time-point and observed clusters of contiguous p-values < 0.05 were compared against the distribution of maximal clusters obtained from the permutations. If the size of the observed cluster exceeded the 95^th^ percentile of the null distribution it was deemed significant. In one case (Fig. 6c) a Pearson correlation coefficient was computed. For (3) the observed CP value was compared to the constructed distribution of CP values and was classified as significant if it exceeded the 95^th^ percentile of that distribution.

#### Calculating time-courses of spike count correlations and choice probabilities

To calculate the time-course of rSC or CP over the course of the trial, we counted all spikes falling into a sliding 100 ms time-window (T) (1 ms bin-size). CPs and rSC values were then calculated and de-trended as defined above, across trials but within a given time-window.

To obtain rSC matrices, we restricted our spike count data to come from independent time-windows (i.e. time-bins) by averaging across time-points centred at t = T/2, 3/2T, 5/2T and so on resulting in 20 time-bins for the trial duration of 2 s. Then, for each site we calculated the rSC between time-bin t_i_ across trials in neuron one (i.e. SU) with the same and any other time-bin t_j_ across trials in neuron two (i.e. MU). All pairwise combinations give the rSC matrix for a site. The average of those matrices gives the rSC matrix for the tested population.

To estimate the time-course of rSC at varying time-lags across the trial, for each time-bin t_i_ we averaged the corresponding leading and lagging correlation values obtained from our rSC matrix (per site). For simplicity, we refer to any rSC values derived in that way as rSCτ, where the τ represents the dynamically chosen number of leading and lagging rSC values making up rSCτ. To investigate correlations at longer timescales, τ was set to four, which corresponds to an effective time lag/lead of about −450 ms to +450 ms, excluding the main diagonal pairs where t_i_ = t_j_ i.e. τ = 0 (i.e. +/−50 ms). We also examined separately the main diagonal, i.e. τ = 0. In this case rSCτ was referred to as instantaneous correlation.

To identify trends in the rSCτ time-courses, we fitted a 2nd order polynomial to the rSCτ values obtained for each time-bin and took the derivative of our fitted function. Although estimated from independent time-bins, this derivative is now available for each time-point. To test whether the rising or decaying rSCτ predicted CP at any time-point in the trial, we calculated the Pearson correlation between derivatives obtained for each site at any time-point with the CP calculated for the SU at the same or any other time-point. Finally, to analyse how trends in rSCτ estimated over the whole trial affect CP time-course, we took the average derivative of our polynomial fits to rSCτ and split our population into sites that showed a generally rising (derivative >0) or decaying (derivative <0) rSCτ time-course. After these steps, we computed the average CP time-courses for each subpopulation.

## Supplementary information

Supplementary Information

## Data Availability

Data and code are available from the last author upon reasonable request.
