# Supplementary material for: Interneuronal correlations at longer time scales predict decision signals for bistable structure-from-motion perception
Source: Sci Rep. 2019 Aug 7;9:11449. doi: 10.1038/s41598-019-47786-1 (PMC6686021; doi:10.1038/s41598-019-47786-1)
Supplement: Supplementary file 1 — Supplementary Information [file 41598_2019_47786_MOESM1_ESM.pdf]

Supplementary information to:

Interneuronal correlations at longer time scales predict decision signals for bistable structure-from-motion perception.

Wasmuht DF[1,2*], Parker AJ[1] and Krug K[1*]

[1] *Department of Physiology, Anatomy and Genetics, University of Oxford, Oxford, OX1 3PT, United Kingdom.*
[2] *Department of Experimental Psychology, University of Oxford, Oxford, OX1 3UD, United Kingdom.*

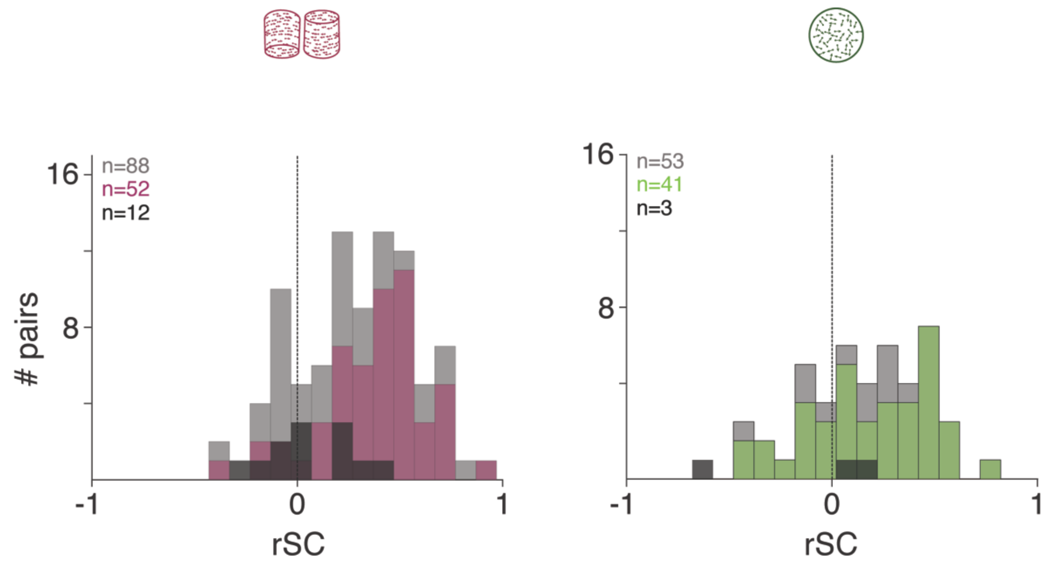

Supplementary Figure 1: Histograms of spike count correlations for the ambiguous cylinder and random dot motion. Grey coloured histograms show stimulus specific rSC distributions estimated from all pairs (i.e. SU/MU pairs) recorded with: the ambiguous cylinder (left panel); random dot motion (right panel). Purple and green coloured distributions indicate respective rSC sub-distributions for pairs with strong and matching SU and MU tuning for cylinder rotation (purple) and motion direction (green). Black coloured histograms represent distributions of stimulus specific rSC values for SU/SU pairs recorded from the same electrode. Dashed vertical lines indicate a rSC of zero.

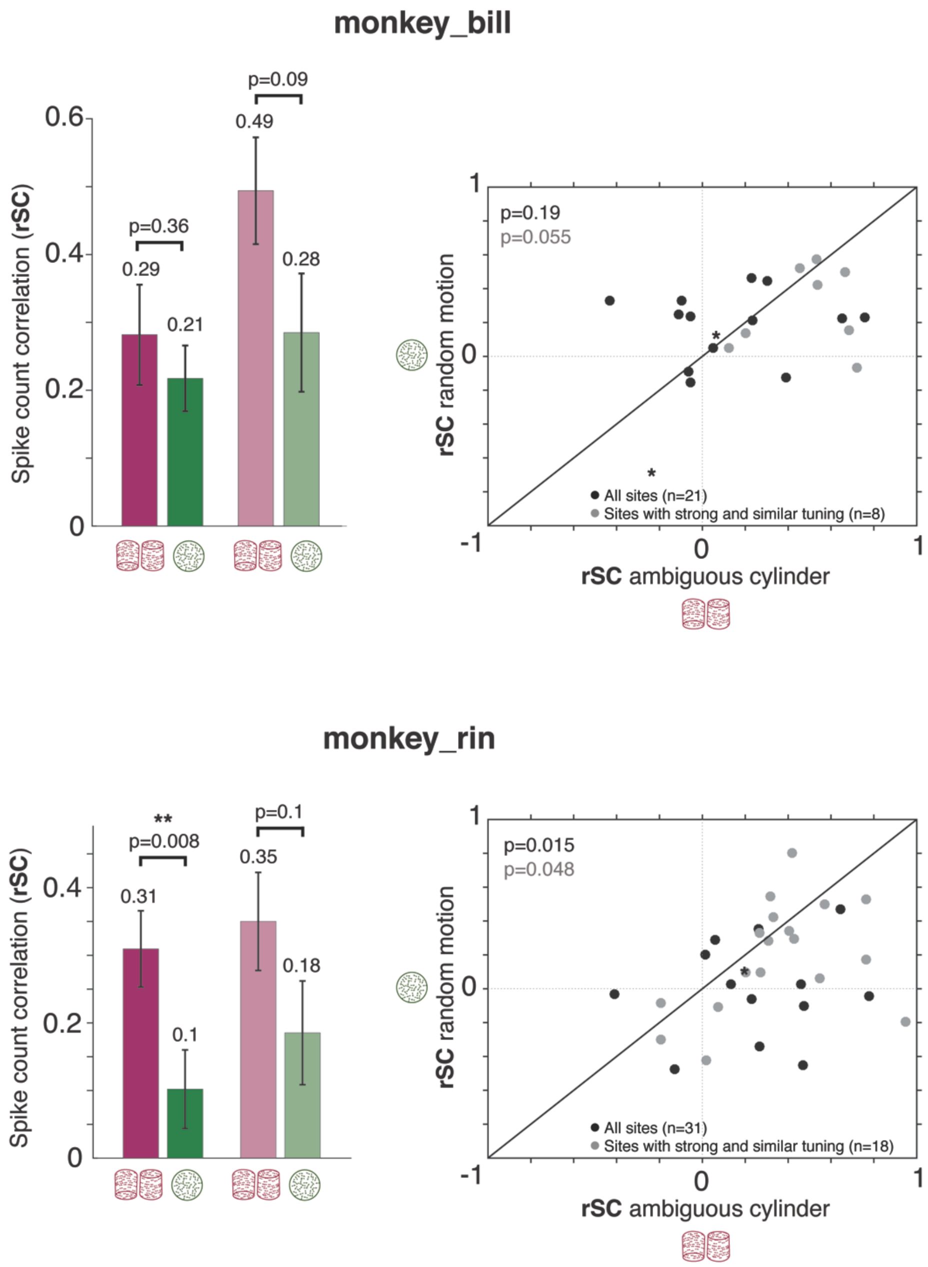

Supplementary Figure 2: Histograms of spike count correlations for the ambiguous cylinder and random dot motion (split by monkey). Conventions are the same as in Fig. 1 in the main text. Left column: Bar plot comparing the average rSC between the two stimuli; ambiguous cylinder (purple) and random motion (green). Bright bars show average values for all sites per animal; pale bars show average values for sites with strong and matching SU and MU tuning. P-values stem from t-tests on Fisher's z transformed rSC values. Error bars indicate s.e.m. Right column: The rSC values for the random motion stimulus (y-axis) are plotted against the rSC values for the ambiguous cylinder (x-axis) from the same sites, for each animal. P-values stem from sign tests.

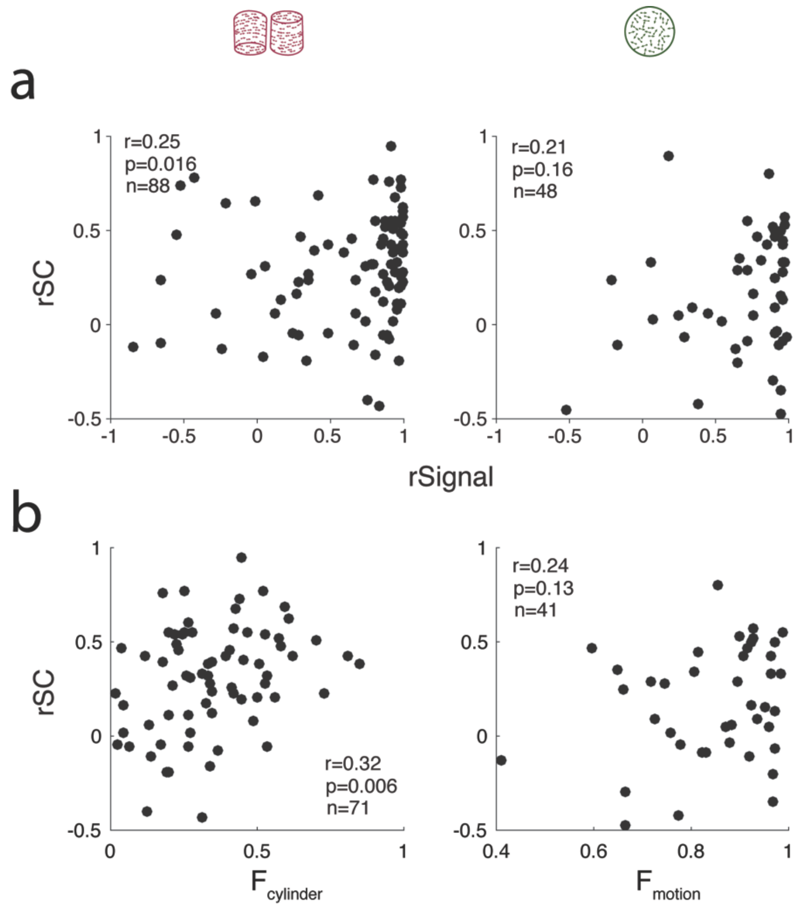

Supplementary Figure 3: Dependence of spike count correlations on tuning similarity and strength. **a)** Scatter plots showing rSC vs. r_{Signal} for all pairs recorded with ambiguous cylinder stimuli (left panel) and/or random motion stimuli (right panel). Each dot represents a recorded site (i.e. MU/SU pair). Correlation coefficients (Spearman rank, r) and associated p-values between rSC and r_{Signal} are displayed. **b)** Scatter plots of rSC for the ambiguous cylinder vs. $F_{cylinder}$ (left panel); rSC for random motion vs. F_{motion} (left panel).

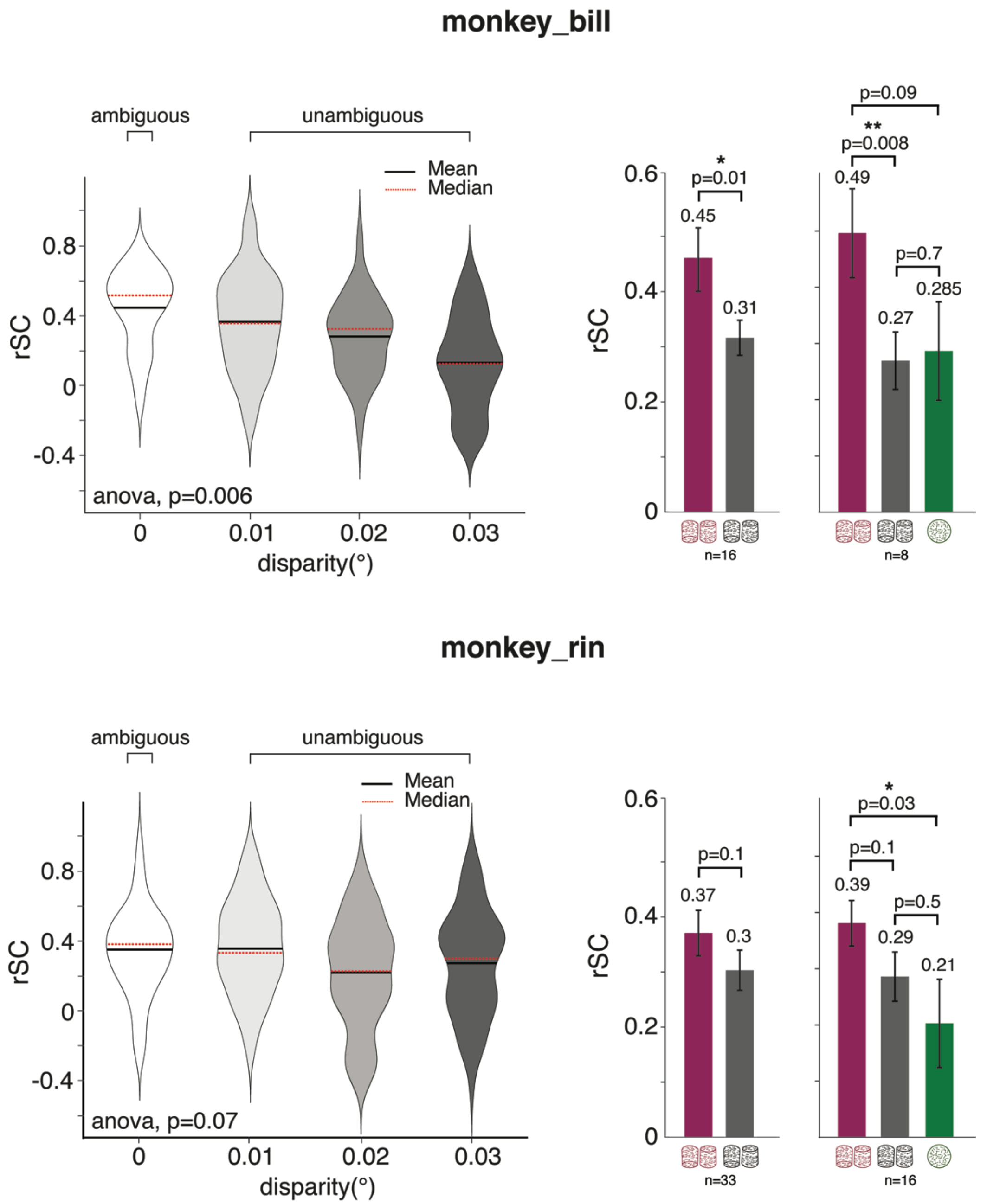

Supplementary Figure 4: Spike count correlations for intermediate cylinder disparities - for each monkey. Same conventions as in Fig. 2 in the main text. Violin plots in the left column show distributions of rSCs for cylinder stimuli with different disparity strengths. P-values stem from one-way ANOVAs on Fisher's-z transformed rSC values. Bar plots in the right column indicate mean rSC values for three stimulus conditions (purple: ambiguous cylinder; grey: unambiguous cylinder; green: random motion). Only sites with strong and matching MU and SU tuning (see Methods) were included. Left panel: Mean rSC values for all sites that were tested with the ambiguous cylinder (zero disparity) and at least one unambiguous cylinder stimulus (i.e. non-zero disparity). Right panel: Mean rSC values for sites that were tested with all three conditions i.e. stimuli. P-values stem from t-tests on Fisher's z transformed rSC values.

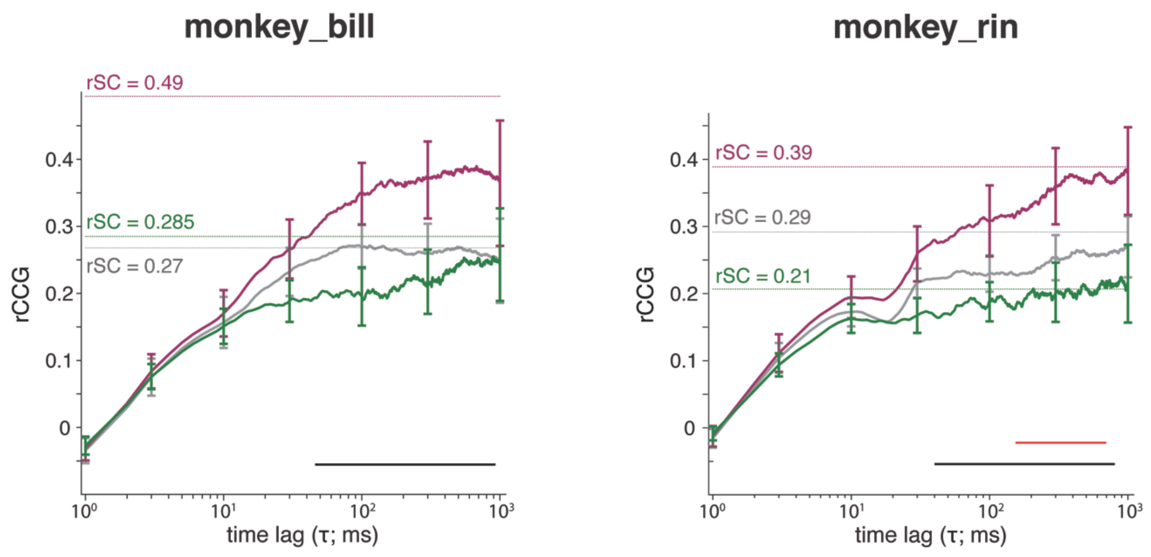

Supplementary Figure 5: Spike time correlations and timescales of correlations – data split by monkey. Same conventions as Fig. 3 in the main text. Average rCCG plotted as a function of the time-lag (τ) i.e. the integration window; for the ambiguous cylinder (purple), the unambiguous cylinder (grey) and the random motion (green) stimuli. Horizontal black and red lines indicate a significant difference between the purple and green and the purple and grey lines, respectively [$p<0.05$, *cluster-based permutation test,* (n=8 in monkey_bill; n=16 in monkey_rin)]. Horizontal dashed lines indicate the corresponding average rSC values. Error bars indicate the s.e.m.

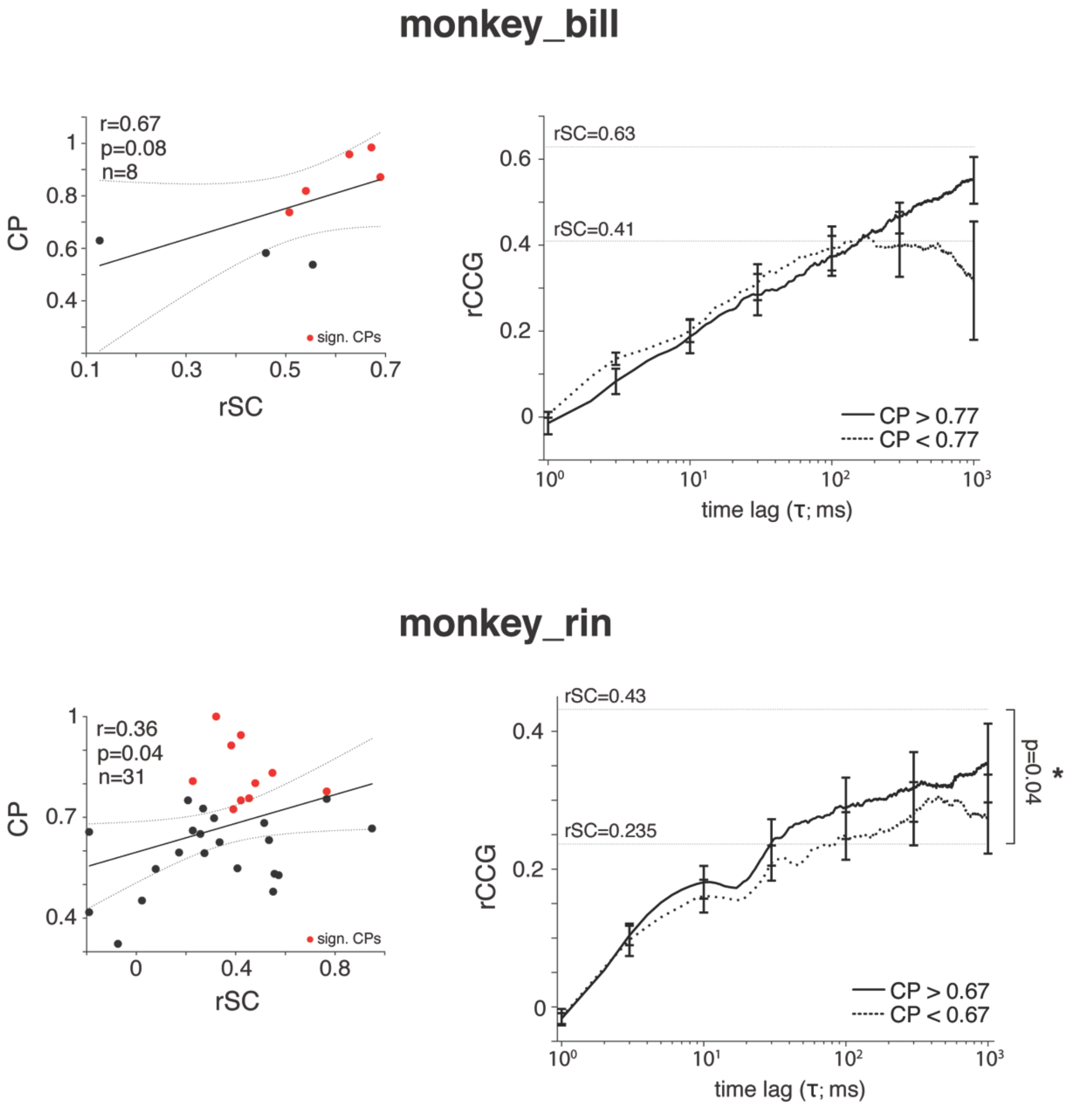

Supplementary Figure 6: Choice probabilities and spike count correlations – data split by monkey. Same conventions as in Fig. 4 in the main text. Left column: Scatter plots of SU CP and SU-MU rSC. Each dot represents a SU that showed strong and matching *cylinder rotation tuning and for which a CP value could be measured. Red dots indicate SUs with a significant CP (p<0.05; cluster based permutation test). Black solid line indicates a linear fit to all data points with 95%* confidence interval (dashed lines) and displayed correlation coefficient and associated p-value stem from the Pearson correlation. Right column: Average rCCG for subpopulations of sites split by the median CP associated with the SU. Error bars indicate the s.e.m. Horizontal lines indicate corresponding average rSC values. P-value stems from un-paired t-test on Fisher'z rSC values.

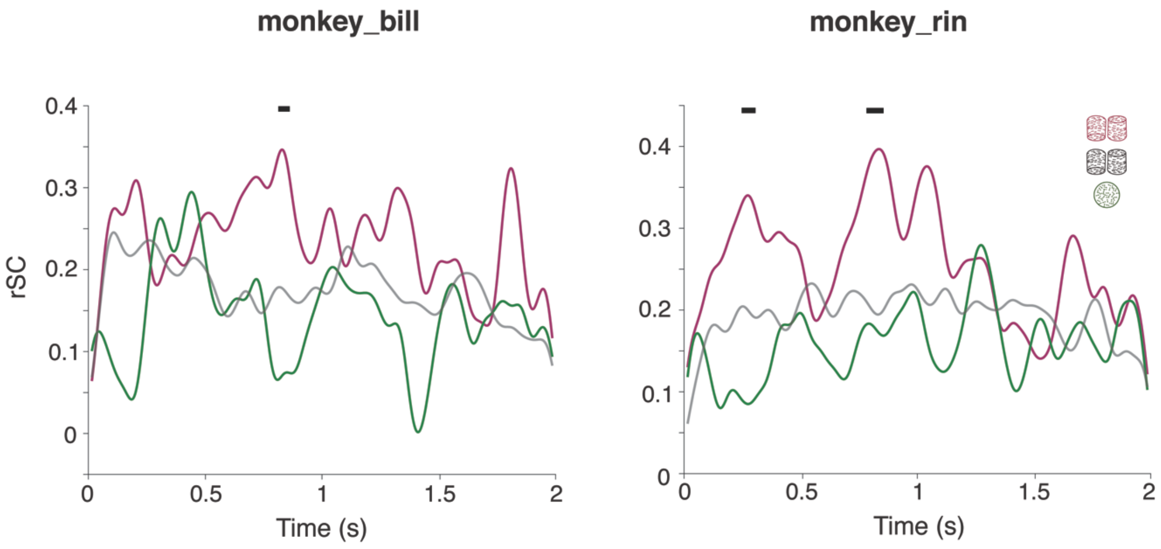

Supplementary Figure 7: Time-course of spike count correlations – data split by monkey. Same conventions as in Fig. 5 in the main text. Average rSC time-courses estimated from a sliding 100ms window. Purple, grey and green lines show the average rSC time-courses for the ambiguous cylinder, the unambiguous cylinder and random motion, respectively. Averages were computed over all sites recorded under all three stimulus conditions (n=8 in monkey_bill; n=16 in monkey_rin). The black horizontal bar indicates a time window with a significant difference between the ambiguous cylinder and the random motion condition ($p<0.05$; *cluster based permutation test*).

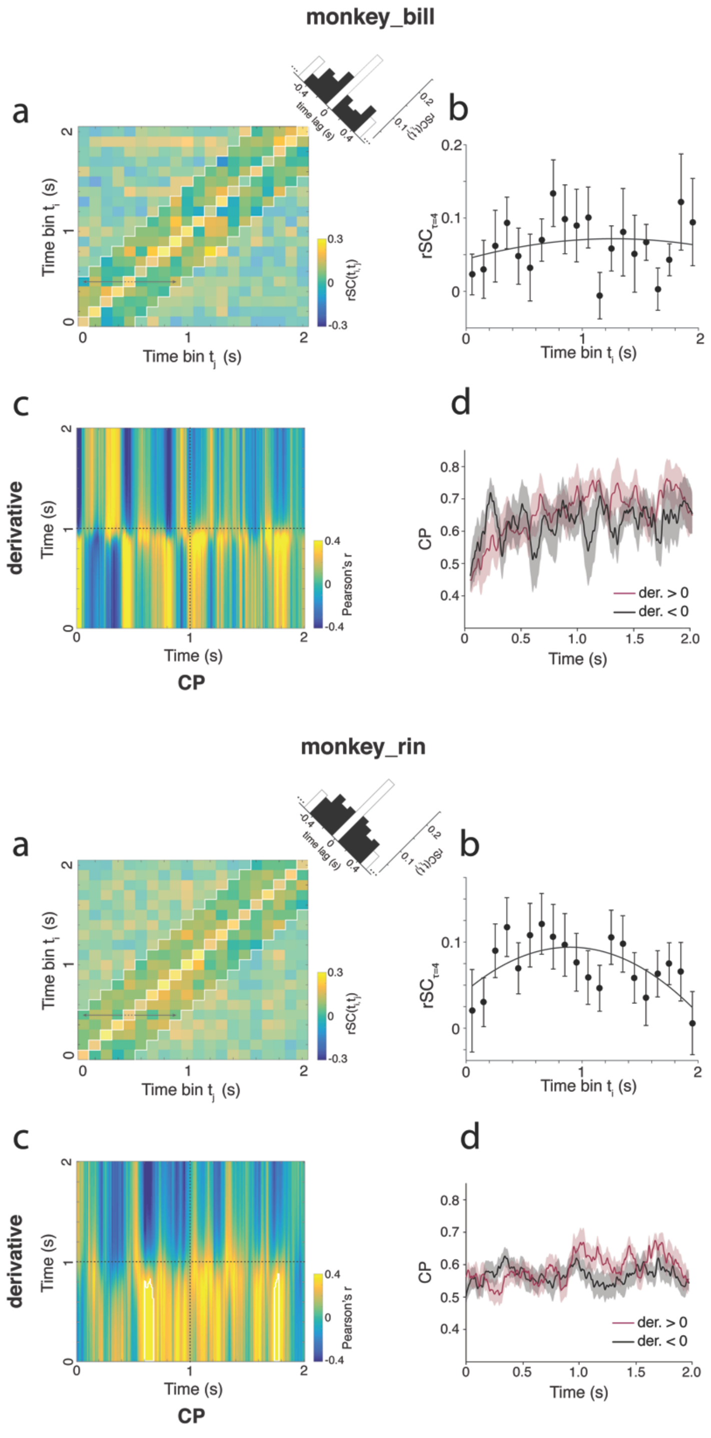

Supplementary Figure 8: Correlation time-course predicts choice probability - data split by monkey. Same conventions as in Fig. 6 in the main text. **a)** Spike count correlation matrices. **b)** $rSC_{\tau=4}$ averaged over all sites are plotted as a function of time-bins t_i. Error bars show s.e.m. The black line indicates a 2nd order polynomial fit to the $rSC_{\tau=4}$. **c)** The matrix for Pearson correlation coefficients between the derivative of the fitted polynomial in b) and CP across recording sites. White contours mark clusters with a significant Pearson correlation (p=0.01; cluster based permutation test). **d)** The CP time-courses averaged across sites with a negative derivative (black; n=5 monkey_bill; n=14 monkey_rin) and positive derivative (purple; n=4 monkey_bill; n=19 monkey_rin). Shaded area indicates s.e.m.

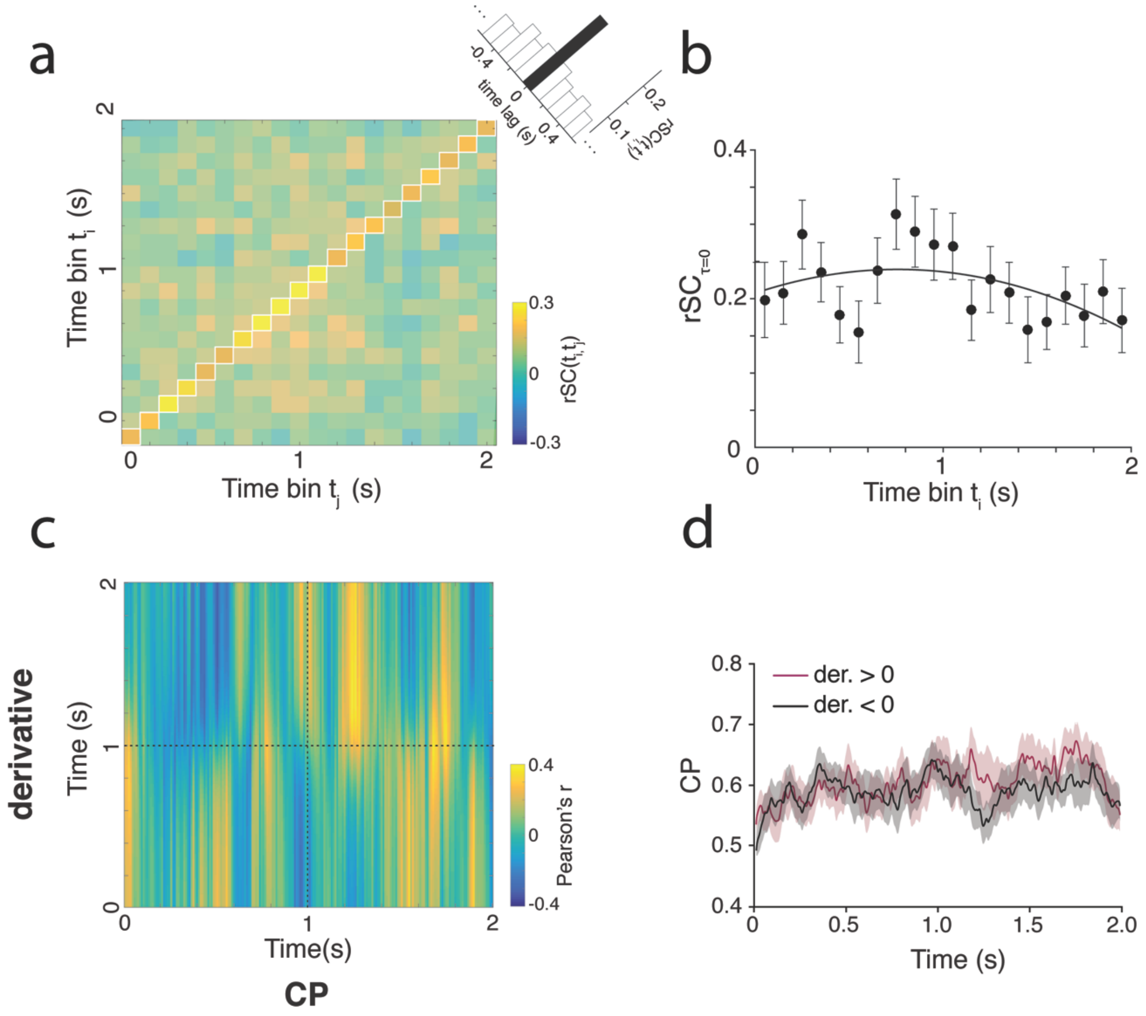

Supplementary Figure 9: Instantaneous correlation time-courses and choice probability. This figure is analogous to Fig. 6 in the text. Now, $rSC_{\tau=0}$ in **b** refers to the instantaneous rSC i.e. the main diagonal highlighted in **a**. Average CP time courses in **d** are comprised of n=24 sites with a derivative < 0 and n=15 sites with a derivate>0.
